# Transient Dimers of Allergens

**DOI:** 10.1371/journal.pone.0009037

**Published:** 2010-02-05

**Authors:** Juha Rouvinen, Janne Jänis, Marja-Leena Laukkanen, Sirpa Jylhä, Merja Niemi, Tero Päivinen, Soili Mäkinen-Kiljunen, Tari Haahtela, Hans Söderlund, Kristiina Takkinen

**Affiliations:** 1 Department of Chemistry, University of Eastern Finland, Joensuu, Finland; 2 VTT Technical Research Centre of Finland, Espoo, Finland; 3 Department of Allergy, Helsinki University Central Hospital, Helsinki, Finland; Massachusetts Institute of Technology, United States of America

## Abstract

**Background:**

Allergen-mediated cross-linking of IgE antibodies bound to the FcεRI receptors on the mast cell surface is the key feature of the type I allergy. If an allergen is a homodimer, its allergenicity is enhanced because it would only need one type of antibody, instead of two, for cross-linking.

**Methodology/Principal Findings:**

An analysis of 55 crystal structures of allergens showed that 80% of them exist in symmetric dimers or oligomers in crystals. The majority are transient dimers that are formed at high protein concentrations that are reached in cells by colocalization. Native mass spectrometric analysis showed that native allergens do indeed form transient dimers in solution, while hypoallergenic variants of them exist almost solely in the monomeric form. We created a monomeric Bos d 5 allergen and show that it has a reduced capability to induce histamine release.

**Conclusions/Significance:**

The results suggest that dimerization would be a very common and essential feature for allergens. Thus, the preparation of purely monomeric variants of allergens could open up novel possibilities for specific immunotherapy.

## Introduction

Allergic diseases, for example, asthma, rhinitis, eczema, and food allergies are reaching epidemic proportions in the world. These type I hypersensitive reactions are based on the formation of immunoglobulin E (IgE) antibodies against, in principle, harmless proteins, allergens. The mature B cells secrete these IgE antibodies, which are able to bind both to allergens but also to high affinity FcεRI receptors on a mast cell or basophil surface. The allergen induces cross-linking of FcεRI-IgE complexes on the cell surface which triggers the granulation of biological mediators like histamine and lipid mediators that cause inflammatory reactions [1]. Today, over 1500 allergens have been identified [2]. Although the allergens are classified as belonging to only 2% of the known protein families with a restricted number of biological functions [3], the factors that make a protein allergenic are largely unknown.

In 2007, we published the first three-dimensional structure of an allergen complexed with an IgE antibody. In this structure, the dimeric β-lactoglobulin (BLG, Bos d 5) from cow's milk is bound to two IgE/Fab fragments. Surprisingly, the IgE-binding epitope of Bos d 5 covered a flat area on the allergen surface, which is unusual because, according to the crystal structures, the majority of known IgG epitopes are located in protruding areas of antigens. The IgE/Fab fragments were bound to the dimeric Bos d 5 symmetrically, allowing, in principle, the cross-linking of FcεRI receptors on the mast cell by the two identical IgE antibodies [4]. This, in turn, would trigger the mast cell granulation. The observation of the possible role of dimerization for the allergenicity of Bos d 5 led us to further investigate how common dimerization is among allergens. In fact, Bos d 5 is a well-studied example of a transient dimer whose dissociation constant (K_d_ = 5 µM) is so high that protein exists as a mixture of monomers and dimers in solution, depending on the concentration of Bos d 5 [5]. Transient dimers are difficult to observe because the dimeric fraction can be negligible at normal cellular concentrations (10–100 nM). However, by colocalization within a cell, concentration may increase locally (to 1 mM) and the interaction between monomers can increase from neglible to substantial [6]. One example of colocalization is the binding of antigens (allergens) on the surface bound antibodies of B cells or effector cells. This would mean that an otherwise weak homodimerization of an allergen may be enough for signal transduction. Dimerization is obviously not a theoretical necessity for allergenicity, monomeric allergens can trigger FcεRI cross-linking if the immune system has developed two different IgE antibodies whose binding sites on the surface of an allergen (epitopes) are not overlapped.

Some examples have been described where oligomerization would increase allergenicity of a protein. Schöll *et al.* have analyzed the role of the dimerization of birch pollen allergen Bet v 1 for cross-linking. Skin tests in Bet v 1 allergic mice were positive with Bet v 1 dimer, but remained negative when the monomer was used. In addition, the monomer was less capable of activating murine memory B cells for IgE production *in vivo*. In this study, a monomeric form of Bet v 1 was prepared with the addition of 4% glycerol [7]. However, the effect of an additive is only temporary because glycerol is gradually diluted and the normal monomer/dimer equilibrium is reached. The influence of a quaternary structure upon the allergenicity and immunogenicity of cockroach allergen Per a 3 has also been investigated. A hexameric form induced a stronger leukotriene release from basophils than the monomeric form. Unfortunately, the preparation of monomeric and hexameric forms of Per a 3 was not described in the paper [8]. Recently, Li *et al.* prepared mutants of the dimeric cockroach allergen Bla g 2. One constructed mutant appeared to be monomeric, based on size exclusion chromatography analysis, and it induced less β-hexosaminidase release from mast cells than the authentic Bla g 2 [9]. However, it has been shown that the trimeric birch pollen allergen Bet v 1 is less allergenic than the native allergen [10]. In this study, the homomer was created by constructing fusion proteins from the monomers and thus the assembly probably differs markedly from native structures.

Starting with the hypothesis that dimerization or oligomerization is a central feature of many allergens, we have systematically investigated the capability of allergens to form dimers, especially transient dimers and the role of dimerization for allergenicity. We did this by i) analyzing all available crystal structures of allergens in the Protein Data Bank, ii) studying experimentally the dimerization of selected allergens in solution by using electrospray ionization mass spectrometry (ESI-MS), and iii) by preparing a monomeric mutants of Bos d 5. We conclude that our hypothesis finds support in the available data.

## Materials and Methods

### Analysis of Crystal Structures

The Protein Data Bank (www.rcsb.org) was used for searching allergen crystal structures. The Allergome data base (www.allergome.org) was exploited to check allergenic nature of found proteins. Found 55 allergen structures were studied by using the PISA server from the European Bioinformatics Institute (www.ebi.ac.uk/msd-srv/prot_int/pistart.html) [11]. PISA uses the crystal data to predict an oligomeric state (quaternary structure). However, the program does not use protein concentration as a parameter, therefore it is not suitable for detecting transient dimers, for example, it estimates that Bos d 5 would be monomeric. In spite of that PISA is useful in this study because it gives protein-protein interfaces and areas of interfaces per monomer (Table 1). In principle, it is difficult to distinguish dimeric interface from crystal contacts. However, true homodimeric complexes are almost always symmetrical having a rotational symmetry axis between monomers [11]. Therefore, we used the symmetry axis as the search criterion. The interface area between the monomers and coordinates for homomers was calculated with PISA. The figures of the homomers were created with the PYMOL program (Delano, W. L. The PyMol Molecular Graphics System, www.pymol.org).

**Table 1:** Crystal structures of allergens.

allergen	reported oligomerization state	oligomerization state in crystal	Monomer molecular weight (Da)	monomer-monomer interface (Å^2^)	Molecules in the asymmetric unit	Space group	PDB-code	source
**Allergens forming symmetric oligomers in crystals, ranked according to the interface area**								
Pru du 11S globulin	6	6	60874	23475	6	P4_1_	3EHK	almond
Ara h 3	6	6	60624	23205	1	P6_3_22	3C3V	peanut
Dau c 1	2	6	16662	4008	4	P4_1_32	2WQL	carrot
Pru p 3	1	4	10120	4010	2	P6_5_22	2B5S	peach
Asp f 6	4	4	23100	3360	4	P2_1_2_1_2_1_	1KKC	Aspergillus fungi
Api m 4	4	8	2860	3200	2	C222_1_	2MLT	honey bee
Che a 3	2	4	9460	2901	4	P2_1_2_1_2	2OPO	Lamb's-quarters
Phl p 7	2	2	8580	2569	2	P2_1_	1K9U	timothy
Gly m lectin	4	4	27830	2380	1	I4_1_22	1SBF	soybean
Rat n 1	4	4	17820	2334	4	P2_1_2_1_2_1_	2A2G	rat (urine)
Phl p 1	2	2	26400	1368	2	P2_1_2_1_2_1_	1N10	timothy
Hev b 2	no report	2	35525	1358	4	P2_1_	3EM5	rubber (latex)
Gal d 2	1	2	45000	1276	4	P1	1OVA	hen (egg)
Hev b 6	1	4	4730	1268	1	P3_1_21	1WKX	rubber (latex)
Fel d 1	2	2	10190	1239	2	P1	2EJN	cat
Bla g 2	1	2	36300	1149	1	C2	1YG9	german cockroach
Equ c 1	2	2	18920	1025	1	P4_3_2_1_2	1EW3	domestic horse
Mala s 13	2	2	13310	994	2	P2_1_	2J23	malassezia fungi
Bla g 4	1	2	14300	842	2	P4_1_	3EBK	german cockroach
Bet v 1	1	2	17490	822	1	C2	1BV1	birch
Bos d 4	2	2	13640	761	6	P4_3_2_1_2	1F6S	domestic cattle
Api m 2	1	2	38390	732	1	P3_2_21	1FCU	honey bee
Phl p 6	no report	2	12100	709	14	P2_1_2_1_2_1_	1NLX	timothy
Der p 2	1	2	14190	673	2	P2_1_22	1KTJ	european house dust mite
Der f 1	1	2	29700	625	3	P4_1_	3D6S	house dust mite
The I lipase	1	2	13310	610	2	P6_1_	1DT3	Thermomyces fungi
Der p 1	1	2	24420	599	2	C2	3F5V	european house dust mite
Hev b 8	no report	2	14410	586	2	P3_2_	1G5U	rubber (latex)
Api g 1	1	2	16940	569	2	C2	2BK0	celery
Gal d 4	1	2	14190	563	1	P4_3_2_1_2	193L	hen (egg)
Sol i 3	1	2	26350	558	2	P6_1_	2VZN	fire ant
Phl p 5	2	2	31570	542	1	C222_1_	1L3P	timothy
Bos d 5	2	2	15620	528	2	P1	1BEB	domestic cattle
Bet v 2	1	2	14630	525	1	P4_1_2_1_2	1CQA	birch
Gal d 3	1	2	75460	522	1	P4_3_2_1_2	1AIV	hen (egg)
Api m 1	1	2	14740	496	1	I4_1_22	1POC	honey bee
Phl p 2	1	2	10560	413	1	P2_1_2_1_2	1WHO	timothy
Per a 4	2	2	16940	403	2	P4_1_	3EBW	american cockroach
Mus m 1	2	2	17820	389	1	P4_3_2_1_2	1MUP	mouse (urine)
Zea m 1	1	2	26290	379	1	C2	2HCZ	maize
Asp o 21	1	2	52600	328	1	P2_1_2_1_2	2GUY	Aspergillus fungi
Jun a 1	1	2	38000	231	2	P2_1_	1PXZ	cedar
Der f 2	1	2	14190	202	2	P1	1XWV	american house dust mite
Mala s 6	1	2	17820	161	1	P4_1_2_1_2	2CFE	malassezia fungi
**Allergens forming monomers in crystals**								
Act d 1	1	1	27940	-	1	P2_1_2_1_2_1_	2ACT	kiwi
Ara t 8	1	1	14266	-	1	P2_1_2_1_2_1_	1A0K	mouse-ear cress
Asp f 1	1	1	16390	-	2	P2_1_	1AQZ	Aspergillus fungi
Bos d 2	1	1	17160	-	1	P2_1_2_1_2_1_	1BJ7	bovine
Car p 1	1	1	23320	-	1	P2_1_	1KHP	papaya
Chi t 1	1	1	14960	-	1	P3_2_	1ECO	midge
Gly m 1	1	1	8359	-	1	P2_1_2_1_2_1_	1HYP	soybean
Mus a	1	1	37400	-	1	P2_1_2_1_2_1_	2CYG	banana
Ves v 2	1	1	38933	-	1	P2_1_2_1_2_1_	2ATM	yellowjacket
Ves v 5	1	1	22440	-	1	P2_1_2_1_2_1_	1QNX	yellowjacket
Zea m 14	1	1	10230	-	1	P2_1_2_1_2_1_	1MZM	maize

### Allergen Materials

Recombinant allergens from birch pollen (Bet v 1a, Bet v 1d, and Bet v 2), timothy pollen (Phl p 6), *Alternaria alternata* fungus (Alt a 1), rubber tree (Hev b 8), apple (Mal d 1), and celery (Api g 1) were obtained from Biomay AG (Vienna, Austria). Native Bos d 5 (nBos d 5) was purchased from Sigma Chemical Co (Billerica, MA, USA). Lyophilized protein materials were dissolved in water and subjected to buffer exchange (10 mM ammonium acetate, pH 6.9) using PD-10 columns (GE Healthcare, Uppsala, Sweden). Alternatively, protein samples were desalted/buffer-exchanged by HPLC (Äktapurifier™; Amersham Biosciences, Uppsala, Sweden) using HiTrap™ columns (GE Healthcare). The samples were subsequently concentrated using Ultrafree-0.5 (5-kDa cut-off) centrifugal filter devices (Millipore, Billerica, MA, USA) and final protein concentrations were determined from the absorbance at 280 nm by using sequence-derived extinction coefficients. The correctness of the amino acid sequences of the recombinant allergens was verified by ESI-MS measurements of the monomeric proteins, performed under denaturing solution conditions (Table S1).

### Construction and Characterisation of the Monomeric Bos d 5 Mutants

For the construction of monomeric Bos d 5 B variant mutant the histidine146 was substituted with a proline (H146P). For the *E. coli* bacterial production the codon optimized cDNA encoding of the recombinant Bos d 5 B variant (rBos d 5 B) was purchased from GenScript. Using this cDNA as a template the nucleotide substitutions of the H146P mutant were done by a PCR cloning. The cDNAs of the rBos d 5 B and rBos d 5 B H146P mutant were cloned into the bacterial expression vector (pKKtac) and transformed into *E. coli* RV308 strain. The rBos d 5 B and its H146P mutant were produced in a 1-liter scale. The recombinant Bos d 5 B and Bos d 5 H146P were secreted as soluble proteins into the periplasmic space which was isolated by osmotic shock. The periplamic fractions were purifed by ionic exchange chromatography using DEAE Sepharose™ Fast Flow (GE Healthcare) and CM Sepharose™ Fast Flow (GE Healthcare) chromatography. In the case of the rBos d 5 B H146P mutant an additional size-exclusion chromatography step (BioGel P-60, Bio-Rad) was required. The samples for ESI-MS were prepared as above. The molecular masses of the produced rBos d 5 B and rBos d 5 B H146P mutant were in agreement with their calculated values (Table S1).

The allergenicity of the nBos d 5 (Sigma), rBos d 5 B and its H146P mutant was analyzed by a histamine release assay. The assay was performed by the passive sensitization of stripped basophils with the serum from a milk (Bos d 5) allergic patient and as a control with the serum from a non-allergic person followed by the subsequent challenge with allergen preparations (RefLab ApS, Copenhagen, Denmark). The induction of the *in vitro* release of histamine from basophilic leukocytes by nBos d 5, rBos d 5 B and its mutant H146P was measured. Each of the three allergens was tested in the passive transfer test as a dose response study with the following concentrations: 0.03, 0.1, 0.3, 1, 3, 10, 30, 100, 300, 1000, 3000, and 10000 ng/ml.

### Mass Spectrometry

All ESI-MS measurements were performed with a 4.7-T hybrid quadrupole–Fourier transform ion cyclotron resonance (FT-ICR) instrument (APEX-Qe™; Bruker Daltonics, Billerica, MA, USA), equipped with a conventional ESI source (Apollo-II™). Desalted allergen samples in 10 mM ammonium acetate buffer (pH 6.9) were directly infused at a flow rate of 1.5 µL/min with dry nitrogen serving as the drying (200°C, 6 mbar) and nebulizing gas. All instrumental parameters were optimized to maintain non-covalent interactions in the gas-phase and to maximize ion transmission at m/z 2000–3000. The same instrumental parameter settings were employed throughout to avoid any bias in the monomer-dimer ratio between different samples. Typically, 500–1000 co-added 128-kword time-domain transients were recorded and processed to 512-kword data prior to fast Fourier transform and magnitude calculation. Mass calibration was done externally with respect to the ions of an ES Tuning Mix (Agilent Technologies, Santa Clara, CA, USA). All data were acquired and processed with the use of Bruker XMASS 7.0.8 software.

## Results

### Analysis of Crystal Structures

**Figure 1:**
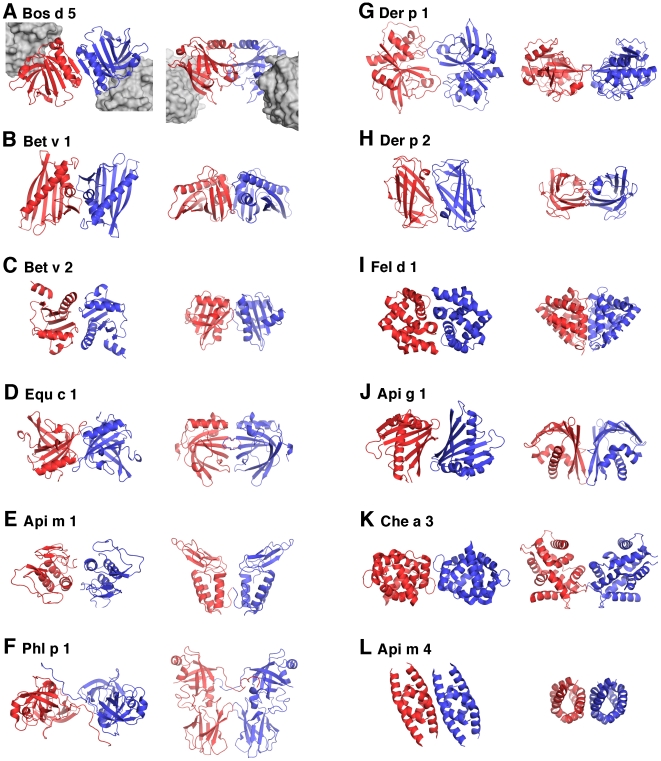
Symmetric homodimers of allergens in crystals. One monomer is shown as a red ribbon model, the second monomer as a blue ribbon model. The dimeric allergen is shown in two orientations. In the left picture the symmetric two-fold axis is towards the viewer, in the right picture the axis is in the same plane as the paper. Fig. **A** shows the crystal structure of a Bos d 5-D1/IgE(Fab) immunocomplex (pdb code 2R56) in which IgE fragments are shown as grey surface models. All structures are drawn to the same scale.

The frequency of dimers was first estimated by studying the packing of monomers in available crystal structures. The Protein Data Bank (www.rcsb.org) contains the coordinates for 55 allergen crystal structures, which were all analyzed. We found that 44 allergens (80%) existed in the crystal forms in which allergens form symmetric homomers, mainly dimers (C2 symmetry) [12]. About half of the dimers were formed by non-crystallographic symmetry (Table 1). The allergens belong to different protein families with different functions, so the dimerization is not based on the same principle. Examples of the allergen dimers are shown in Figure 1.

The interface area between monomers varies considerably, from 161 to 23475 Å^2^. The allergen oligomers in which the monomer-monomer interface area is about 1000 Å^2^ or more can be considered to be quite permanent [5], although many of them have been considered to be essentially monomeric in the literature. For instance, the peach allergen Pru p 3 forms tetramers in the crystal with a very large interface area (4010 Å^2^), but according to the gel-filtration results it was assumed to be monomeric [13]. Bee venom Api m 4 (melittin) forms the obligate tetramer with pseudo-222 symmetry (interface 2610 Å^2^). In addition, there is a 2-fold symmetry axis (interface 590 Å^2^) between tetramers in crystal which suggests that the Api m 4 is able to create even larger assemblies [14]. Pollen allergen Che a 3 forms an obligate dimer [15] (interface 2598 Å^2^) which packs (303 Å^2^) symmetrically with the second dimer forming a tetramer. Cockroach allergen Bla g 2 was described as a monomer in 2005 [16], although it is able to form a symmetric dimer (interface 1149 Å^2^) in a crystal. In 2008, it was described as a dimer in the immunocomplex structure [9].

When the monomer-monomer interface area is less than 1000 Å^2^ the dimers are supposed to be transient [5]. The birch pollen Bet v 1 is one of the best characterized allergen. The crystal structure of Bet v 1 was published in 1996 and the allergen was described as a monomer [17]. However, Bet v 1 monomers form a clear symmetric dimer in this crystal (interface 822 Å^2^). Interestingly, the hypoallergenic variant of Bet v 1 crystallized as a monomer [18] and Bet v 1 also exist as a monomer in the Bet v 1 -IgG/Fab immunocomplex crystals [19], and in this case the binding of a Fab fragment would also prevent the formation of a Bet v 1 homodimer. The bee venom major allergen Api m 2 (hyaluronidase) crystallized in two space groups. The allergen was described as a monomer [20], although our analysis revealed the existence of a symmetric dimer in a more symmetric space group (interface 732 Å^2^). The house dust mite allergen Der p 2 has also been described as a monomer [21], although two molecules in the asymmetric unit form the symmetric dimer (interface 673 Å^2^). The monomers of a major celery allergen Api g 1 allergen [22] form two different dimers in crystal. In the first form, a β-sheet creates the interface (569 Å^2^) and in the second form a long α-helix creates the interface (482 Å^2^), these are both different when compared to the monomer-monomer interface of structurally similar Bet v 1. Hen egg white lysozyme (Gal d 4) forms two different symmetric dimers in crystals (563 and 386 Å^2^). The protein is normally considered to be monomeric in spite of the fact that there is evidence that it forms a dimer at least in higher salt concentrations [23]. On the other hand, timothy grass pollen allergen Phl p 5 has been described as a dimer [24], although the interface area is only 542 Å^2^. The widely studied bovine milk allergen, Bos d 5 is generally considered to be a dimer in spite of the relatively small interface area 528 Å^2^. The allergen also exists as a dimer in the IgE/Fab-immunocomplex structure [4]. Birch pollen profilin (Bet v 2), described as a monomer [25], has a monomer-monomer interface of the same size (525 Å^2^). It has been supposed that a monomeric form is predominant in solution when the interface is smaller than 450 Å^2^ [5]. There are eight crystal structures in which an allergen forms a symmetric dimer in crystal with such a small interface. From these, the mouse urinary allergen Mus m 1 has been previously described as a dimer (interface 389 Å^2^) [26]. According to the gel filtration, Per a 4 from american cockroach was suggested to be dimeric [27]. In crystal, it forms an asymmetric dimer (663 Å^2^) and a symmetric dimer (403 Å^2^).

### Preparation of rBos d 5 and rBos d 5 Mutants

**Figure 2:**
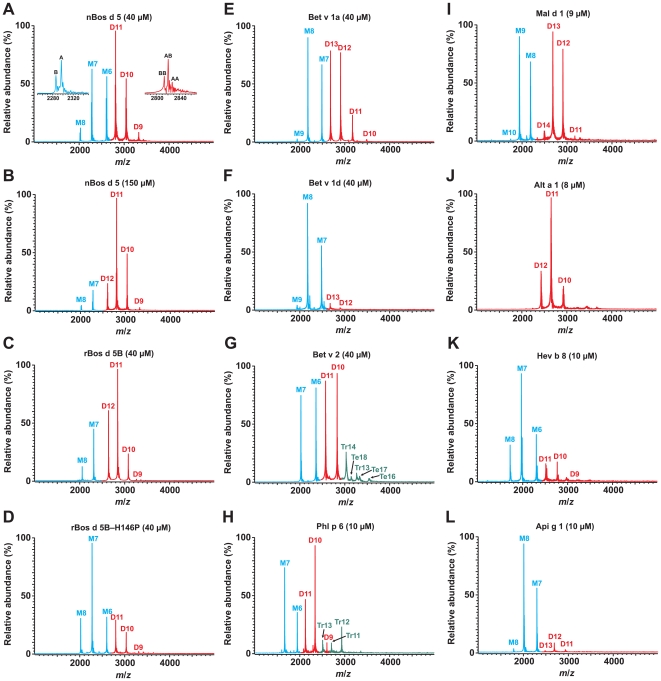
ESI FT-ICR mass spectra of allergens measured in 10 mM NH_4_OAc (pH 6.9) at varying allergen (monomer) concentrations. Numbers indicate different protein ion charges and the letters M (and color blue), D (red), Tr (green), and Te (green) refer to the signals from a monomer, dimer, trimer, and tetramer, respectively. Note that some monomer and dimer charge states overlap in the spectra. The left inset in A shows the magnification of monomer charge state 7+, the right inset the dimer charge state 11+.

To test the influence of dimerization for allergenicity, we prepared a monomeric mutant H146P of Bos d 5 B (variant B) according to Sakurai *et al.* [28]. The rBos d 5 B and its H146P mutant were produced in *E. coli* as soluble proteins to the periplasmic space and purified with chromatographic steps. The anti-Bos d 5 D1 IgE/Fab fragment recognized the recombinant forms demonstrated by a competitive ELISA in which the binding of the D1 IgE/Fab fragment was specifically inhibited to immobilized nBos d 5 by increasing amounts of the rBos d 5 B and rBos d 5 B H146P preparations indicating that these recombinant rBos d 5 B proteins were correctly folded (data not shown) as verified also by the MS analysis (Figure 2).

### Probing Oligomeric State of Allergens by Mass Spectrometry

The formation of allergen oligomers in solution was analyzed by native mass spectrometry with the use of ESI FT-ICR mass spectrometry. Native mass spectrometry refers to an emerging methodology in which proteins are analyzed in their native state thus preserving weak non-covalent interactions in the gas-phase [29]. Although the FT-ICR technique is not considered an ultimate choice for the analyses of large macromolecular protein complexes, we have previously shown that such complexes can also be analyzed by this technique [30], provided that a considerable attention has been paid on the optimization of some critical instrumental parameters. Figure 2 shows ESI FT-ICR mass spectra for selected allergens, measured in 10 mM ammonium acetate (pH 6.9) buffer, at varying protein (monomer) concentration.

In the case of Bos d 5 allergens (Figure 2A-D), the protein concentration of 40 µM was chosen based on the reported K_d_ value (5 µM) for nBos d 5 [28]. Theoretically, at that concentration nBos d 5 exists as a mixture of monomers and dimers with a concentration ratio of 8.8 to 15.6 µM, respectively. Indeed, a mass spectrum of nBos d 5 at 40 µM (Figure 2A) reveals the presence of both monomers (peaks marked in blue) and dimers (peaks in red), with a slightly higher relative abundance for the dimeric form, consistent with the theoretical calculation. To demonstrate that mass spectral abundances reflect the true thermodynamic equilibrium in solution, nBos d 5 was also measured at considerably higher concentration. The measurement at 150 µM (Figure 2B) reveals a considerable increase in the amount of the dimer as compared to the monomer, as expected.

An interesting finding was made considering the two naturally occurring Bos d 5 variants, A and B. These variants differ at positions 64 (Asp/Gly) and 118 (Val/Ala). An inset in Figure 2A (monomer charge state 7+) indicates that the abundance ratio of these two forms in the monomeric protein is about 65 and 35%, based upon the mass spectral peak heights (averaged over all detected charge states). However, the other inset (dimer charge state 11+) shows that the dimeric protein consists of three species AA, AB, and BB, with experimental abundance ratios of 18, 54 and 27%, respectively. If one assumes there is the same K_d_ value for both the variants, then the abundance ratios would be 42, 46 and 12% for AA, AB and BB, respectively. This suggests that the two variants have different K_d_ values (lower for BB than for AA dimer), with an average value of ∼5 µM. In addition, a mass spectrum of rBos d 5 B (Figure 2C) shows slightly higher abundance for the dimer than for the monomer as compared to nBos d 5, which further confirms the lower K_d_ for BB than for AA. In contrast, a mass spectrum of the rBos d 5 B H146P mutant (Figure 2D) shows a considerably decreased amount of dimer as compared to nBos d 5 or rBos d 5 B, indicating a major effect of this mutation on the monomer-dimer ratio.

Interesting findings were also made with the major birch pollen allergens (Figure 2E-G). A mass spectrum of Bet v 1a at 40 µM (Figure 2E) also revealed the presence of a large amount of protein dimer, consistent with a previous dynamic light scattering study [7]. However, a mass spectrum of the hypoallergenic variant Bet v 1d (Figure 2F) at the same concentration exhibited only a very small amount of dimer. We also analyzed another birch pollen allergen, Bet v 2, which is considered to be as a monomer in solution. However, a mass spectrum of Bet v 2 at 40 µM (Figure 2G) revealed the presence of a considerable amount of protein dimer. In addition, small amounts of protein trimer and tetramer were also detected.

Other interesting allergens from different sources were also analyzed (Figure 2H-L). A major timothy pollen allergen Phl p 6 (Figure 2H) revealed the existence of protein dimer already at a concentration of 10 µM. A protein trimer was also detected. A mass spectrum of a major apple allergen (Mal d 1) also shows a considerable amount of protein dimer present at 9 µM (Figure 2I). A fungal allergen from *Alternaria alternata* (Alt a 1) represents so-called permanent dimer due to a presumed disulfide linkage between naturally occurring 14.5- and 16-kDa subunits [31]. Indeed, a mass spectrum of a recombinant Alt a 1, measured at 8 µM (Figure 2J), shows only peaks of a 29-kDa protein dimer. However, under denaturing (but not reducing) solution conditions Alt a 1 effectively dissociated into its monomeric form (data not presented), providing evidence that its dimerization is not mediated by the disulfide bond. Two other allergens (Hev b 8 and Api g 1) also showed a presence of protein dimers at 10 µM (Figure 2K, L). On the basis of crystal structure it had been suggested that Api g 1 would be monomeric [22].

### The Allergenicity of the rBos d 5 B and rBos d 5 B H146P Mutant

**Figure 3:**
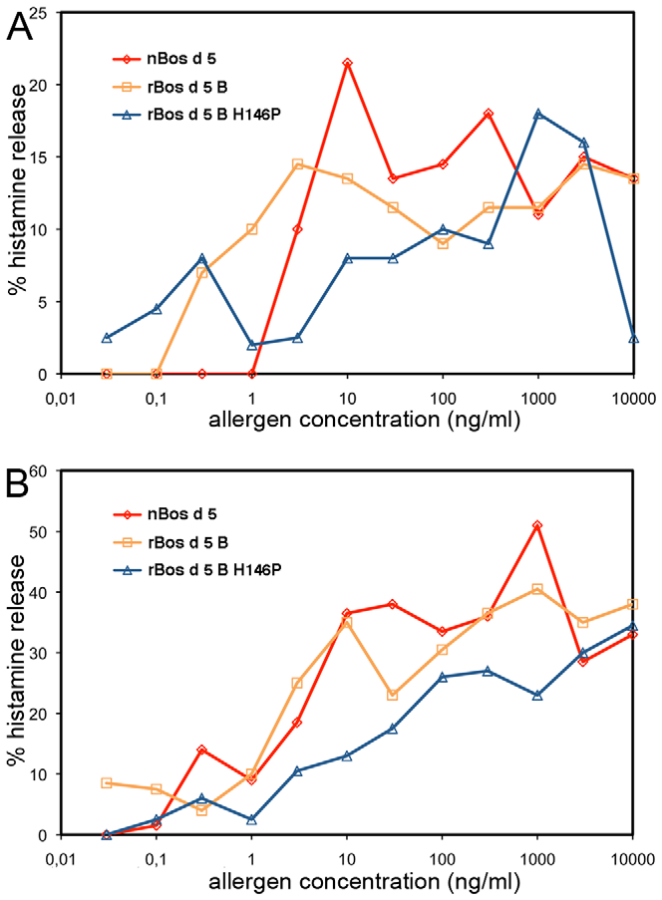
Histamine release experiments with nBos d 5, rBos d 5 B variant, and rBos d 5 B variant H146P mutant. Released histamine was measured after the passive sensitization of stripped basophils from two donors (A; right panel and B, left panel) with the serum from a milk (Bos d 5) allergic patient and purified allergens in a concentration range of 0.03-10000 ng/ml (x-axis, logarithmic scale) in duplicate measurements. The percentage of histamine released into the supernatant is shown on the y-axis (a meanvalue of the duplicate measurments).

The allergenicity of the rBos d 5 B and its mutant H146P was analyzed by *in vitro* histamine release from stripped and sensitized basophils of two different donors. The histamine release induced by the Bos d 5 B mutant H146P, that was shown by the MS analysis to exist mainly as a monomer, show decreased histamine release capacity when compared to the recombinant Bos d 5 (B variant) and native Bos d 5 (AB variants) (Figure 3B).

## Discussion

### The Significance of Crystal Structures

We found that 44 allergen crystal structures out of 55 in the Protein data bank showed the presence of a symmetric oligomer (mainly dimer). Traditionally, the observation of higher order assemblies in crystals have been regarded as a crystallization artifact except if experiments also indicate their presence in solution. However, this view does not take into consideration that the formation of homomers may be dependent on the concentration. It has been estimated that colocalization in cells may increase the concentration of protein locally by up to 1 mM [6]. In a typical crystallization experiment, the concentration of protein is about 10 mg/ml which corresponds to a 0.5 mM concentration for a 20 kDa protein. The observed dimers in crystals could thus be biologically - and in this case - immunologically relevant. The proportion of symmetric homomers among allergens is very high, giving evidence that the capability to form symmetrical oligomers in crystals is a very common property among allergens. Eleven allergens exist as monomers in crystals. This may be due to the crystallization conditions (pH, precipitants) or that a monomeric form has been crystallized. For example, allergens Bos d 4, Api m 2, Gal d 4, Asp o 21 existed as a monomer or a dimer in different crystal forms.

The size of the monomer-monomer interface area is not the only parameter which affects monomer-dimer equilibrium. The geometrical (planarity) and physicochemical (hydrophobicity, hydrogen and ionic bonds) properties are also important. These all have an effect on the dissociation constant (K_d_) of the dimer, which can not be deduced directly from the structure. However, the size of the monomer-monomer interaction area gives some starting point for us to evaluate the stability of the dimer. When the interface is about 1000 Å^2^ or more, the dimers can be considered quite permanent. 17 allergens have such a high interface area and almost all have been considered to be homomers in the literature. 24 allergens have a monomer-monomer interface area between 300 to 1000 Å^2^ suggesting that the dimers would be transient. As would be expected, most of these proteins have been considered to be monomers in the literature. There are three structures in which the interface area is very small (160 to 300 Å^2^) and it is tempting to speculate that the dimerization would also enhance the cross-linking capacity in these cases.

### The Significance of Mass Spectrometric Measurements

Mass spectrometric data of allergens and hypoallergens clearly suggest that allergenicity is related to a capability to form dimers or in some cases higher oligomers. Thus, ESI-MS analysis of intact protein would allow a convenient way to test the allergenicity potential of proteins by direct detection of their oligomeric state in solution. The determination of the oligomeric state of proteins is normally based on size-exclusion chromatography, ultracentrifugation, X-ray scattering or dynamic light scattering. The measurement is challenging, however, if the quaternary structure is not stable, leading to an equilibrium between monomers and dimers (or higher oligomers). In fact, the dissociation constant has been determined for only one allergen, Bos d 5 [28]. ESI-MS allows simultaneous and direct analysis of all protein species (monomers, dimers and higher oligomers) in solution. Therefore, the method is very suitable for detecting transient oligomer formation of proteins in solution. Unlike other biophysical and biochemical techniques, ESI-MS directly provides stoichiometric information about the non-covalent interaction, thus making it a superior technique [29].

### Hypoallergens

In nature many allergens exist in several variants which often have different allergenic potential. Hypoallergenic variants are especially potential candidates for immunotherapy. Recently, IgE responses for native birch pollen allergen Bet v 1 and two variants Bet v 1d (Bet v 1.0401; 96% amino acid residue identity) and Bet v 1l (Bet v 1.1001; 94% identity) have been studied. Variants were considered to be hypoallergens because they were poor inducers of a mediator release and in addition Bet v 1d also induced a high IgG_4_ response [32]. Bet v 1l crystallized as a monomer [18] probably because N28K mutation could prevent the formation of Bet v 1 like dimer. Also Bet v 1d variant crystallized as a monomer [33]. In addition, our mass spectrometry analysis (Figure 2) suggests that Bet v 1d is monomeric in solution. The reason for a reduced dimer formation of Bet v 1d is less clear because of a lack of mutations on the putative monomer-monomer interface. However, it is possible that some mutations in the hydrophobic core (especially F30V) have slightly modified the structure of the monomer-monomer interface and led to the reduced dimer formation.

### Implications for Allergenicity and Immunogenicity

Symmetry is commonly observed in biological systems. Good examples are viruses which may be very symmetrical objects and which are also targets of our immune system. On the other hand, signal transduction is also often based on symmetry, namely dimerization of cell surface receptors [34]. In this respect, it might not be surprising that so many allergens are able to form symmetrical structures. The formation of higher order assemblies of antigens at higher concentrations might be quite common and generally contribute to immunogenicity [35]. So far, the prediction of the allergenicity of proteins is mainly based on amino acid sequence similarity with known allergens [36]. Our results suggest the analysis of the allergenic potential of proteins should also include analysis of oligomer formation. However, oligomerization alone is not enough for allergenicity, the antigen must also include specific structural features, for example, such as suggested flat surface which render it allergenic [4]. In addition, according to this study, allergenicity would also depend on the concentration. At low concentrations, the allergen is “inactive” because it exists as a monomer. When the concentration is higher, the allergen is “active” as a dimer. If the formation of stable or transient symmetric homodimers is reduced and the allergen would exist only in the monomeric form, this would offer possibilities to develop a new type of hypoallergens: monomeric variants of allergens. The monomeric variants would have advantageous properties in the relation to specific immunotherapy or allergy vaccination. The monomeric allergens would not trigger mast cell or basophil degranulation and they would compete with native allergens to bind to IgE antibodies. Consequently, larger amounts of monomeric variants could be used as an allergy vaccine with reduced side-effects to induce production of protective IgG antibodies.

## Supporting Information

Table S1 Calculated and measured masses for the selected (monomeric) allergens.
(0.05 MB PDF)
