# Supplementary material for: Transient Dimers of Allergens
Source: PLoS One. 2010 Feb 5;5(2):e9037. doi: 10.1371/journal.pone.0009037 (PMC2816702; doi:10.1371/journal.pone.0009037)
Supplement: Table S1 — Calculated and measured masses for the selected (monomeric) allergens. (0.05 MB PDF) [file pone.0009037.s001.pdf]

Table S1. Calculated and measured masses for the selected (monomeric) allergens

Allergen	MW(calcd) Da[1]	MW(meas) Da[2]
nBos d 5 (A/B)	18 362/18 276	18 362/18 276
rBos d 5B	18 276	18 276
rBos d 5B-H146P	18 236	18 236
Alt a 1[3]	14 664	15 237
Mal d 1	17 567	17 566
Dau c 1	17 338	17 337
Api g 1	16 188	16 188
Hev b 8	13 903	13 900
Phl p 6	11 790	11 789

[1] Calculated (average) mass based on the protein sequence (SwissProt, EMBL)

[2] Measured mass, averaged over observed charge state distributions (average uncertainty ± 1 Da). Mass measurements were performed with ESI FT-ICR instrument, employing denaturing solution conditions (MeCN/H2O/HOAC, 49.5:49.5:1.0, v/v) in which all allergens dissociated into monomers.

[3] The reason for the difference between calculated and measured masses is unknown; It is not apparent, however, if the expression vector construct of recombinant Alt a 1 from Biomay differs from that predicted based on the SwissProt sequence.
